# Mucosal BCG Vaccination and Immune Layering: Route-Dependent Programming of Immunity at the Respiratory Interface

**DOI:** 10.3390/vaccines14080667

**Published:** 2026-07-31

**Authors:** Yukihiro Shibuya, Miyu Sakai, Hideyasu Kiyohara

**Affiliations:** Research and Development Department, Japan BCG Laboratory, 3-1-5 Matsuyama, Kiyose 204-0022, Tokyo, Japan

**Keywords:** Bacille Calmette–Guérin (BCG), tuberculosis, mucosal immunity, trained immunity, tissue-resident memory T cells, vaccination route, immune layering

## Abstract

*Mycobacterium bovis* Bacille Calmette–Guérin (BCG), the only licensed vaccine against tuberculosis, provides inconsistent protection against pulmonary tuberculosis, reflecting an incomplete understanding of how vaccine-induced immunity is organized within tissues. Emerging evidence indicates that the route of vaccination is not merely a technical variable but a critical determinant of immune programming. Whereas parenteral BCG primarily elicits systemic immune responses, mucosal delivery reprograms immunity at the respiratory interface by promoting localized trained innate immunity, tissue-resident memory T (T_RM_) cells, and early containment of infection. In this review, we propose an integrated framework of “immune layering,” in which protection emerges through the coordinated interactions of epithelial regulation, trained innate immunity, tissue-resident adaptive memory, regulatory homeostasis, and systemic immune support across spatial and temporal scales. Within this framework, trained innate immunity serves as an initial conditioning layer that shapes subsequent adaptive differentiation, whereas epithelial- and microbiota-associated regulatory networks establish the tissue context in which immune responses are initiated, organized, and maintained. Importantly, effective mucosal immunity depends on a dynamically regulated equilibrium rather than maximal immune activation. The dissociation between enhanced early pulmonary immune responses and limited long-term protection underscores the influence of tissue-specific regulatory constraints and environmental context on vaccine efficacy. This framework redefines correlates of protection by identifying the vaccination route and tissue-level immune organization as fundamental determinants of protective immunity, thereby providing a conceptual foundation for the rational development of next-generation mucosal tuberculosis vaccines.

## 1. Introduction

Tuberculosis (TB) remains one of the leading causes of death from infectious diseases worldwide, despite the availability of *Mycobacterium bovis* Bacille Calmette–Guérin (BCG), the only licensed vaccine against TB [1,2]. Although BCG has been used for more than a century, the immunological mechanisms underlying its variable and context-dependent protective efficacy remain incompletely understood. Beyond its established role in inducing pathogen-specific adaptive immunity, BCG has emerged as a potent inducer of trained immunity, a form of innate immune memory mediated by epigenetic and metabolic reprogramming of innate immune cells [3,4,5,6]. This functional reprogramming promotes heterologous protection against unrelated pathogens and enhances innate immune responsiveness over time [6,7], challenging the traditional paradigm that vaccine-induced protection is mediated exclusively by antigen-specific adaptive immune memory. Accumulating evidence indicates that the route of vaccination is a critical determinant of these immunological outcomes. Human studies have demonstrated that different routes of BCG administration elicit distinct systemic and mucosal transcriptional programs, indicating that vaccination route shapes the spatial organization and functional architecture of immune responses rather than merely influencing their magnitude [8,9]. This distinction is particularly relevant because *Mycobacterium tuberculosis* (Mtb) infection is initiated at the respiratory mucosa [10]. However, most mechanistic insights into BCG-induced immunity have been derived from parenteral vaccination, which predominantly elicits systemic immune responses and may not fully engage the immune networks operating at mucosal surfaces. Consequently, the earliest host–pathogen interactions at the site of natural infection remain incompletely represented in conventional models of vaccine-induced immunity.

Renewed interest in mucosal BCG delivery, particularly via intranasal and intratracheal administration, has begun to address this limitation [11,12]. These approaches induce immunological features that are less prominent following parenteral vaccination, including rapid local immune activation, generation of tissue-resident memory T (T_RM_) cells, and coordinated interactions among epithelial, innate, and adaptive immune compartments within the respiratory tract [13,14,15,16]. Importantly, immune reprogramming induced by BCG alone is insufficient to ensure durable protection. Clinical studies of BCG revaccination have demonstrated variable and context-dependent protective effects, suggesting that protection depends not only on immune response magnitude but also on the quality and localization of immune responses [17]. Collectively, these observations identify the vaccination route as a fundamental determinant of immune outcomes by influencing the localization, integration, and persistence of protective immune responses. Accordingly, effective immunity against TB is likely to depend on the establishment of coordinated immune networks at the respiratory mucosa rather than on systemic immunity alone.

In this review, we synthesize current knowledge of BCG-induced mucosal immunity, with an emphasis on route-dependent immune programming, the coordination of innate and adaptive immune responses, tissue-resident memory formation, and regulatory influences exerted by epithelial cells and the respiratory microbiota. Building on these concepts, we propose a unifying framework of “immune layering” to explain how the spatial and temporal organization of mucosal immunity can be harnessed to guide the rational design of next-generation TB vaccines.

## 2. Route-Dependent Immune Programming by BCG Vaccination: Why Mucosal Delivery Matters

**Figure 1:**
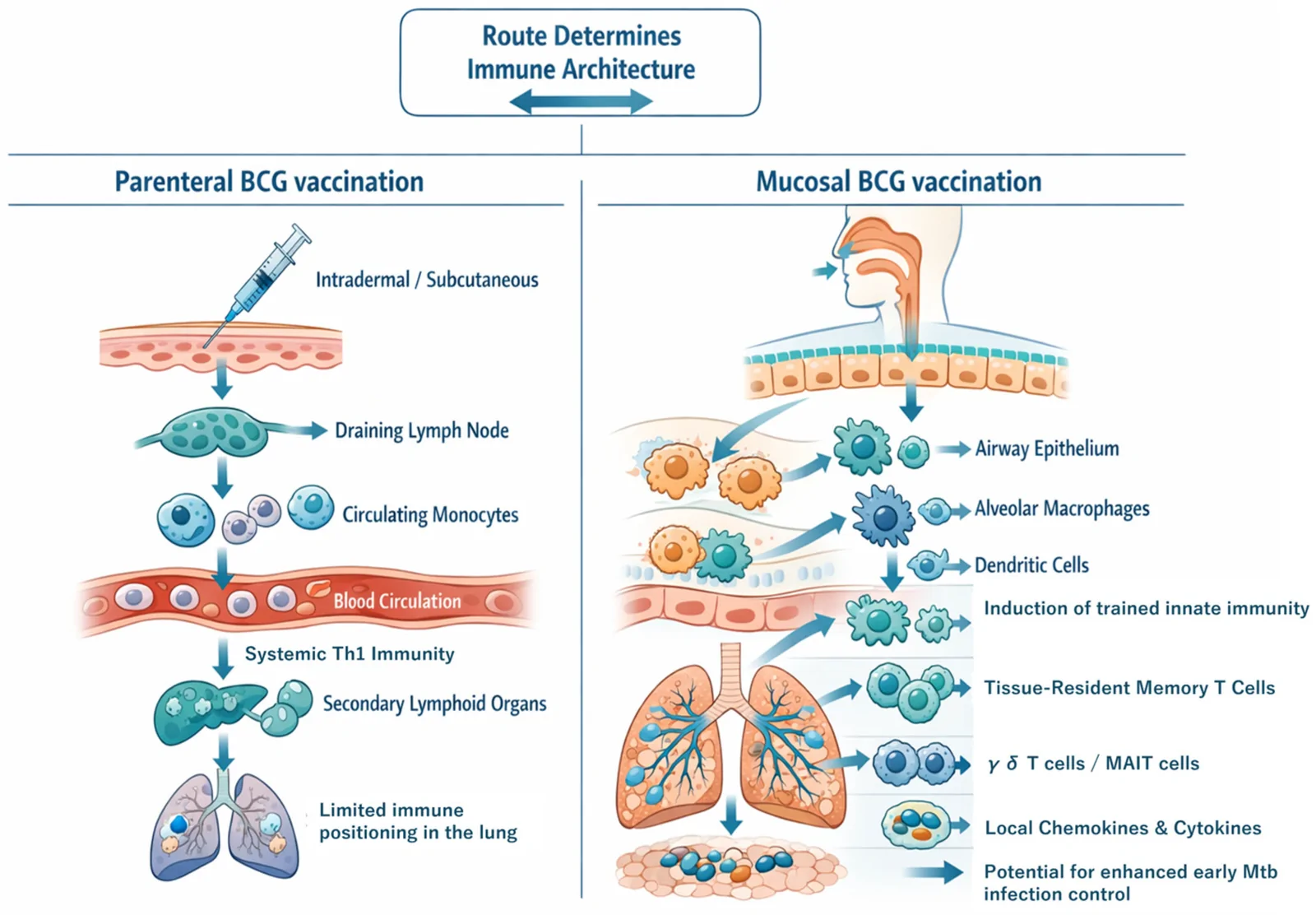
Route-dependent immune architecture induced by BCG vaccination. This schematic illustrates how the route of Bacille Calmette–Guérin (BCG) administration influences the spatial organization of vaccine-induced immunity. (**Left**) (Parenteral BCG vaccination): Intradermal or subcutaneous vaccination primarily induces systemic immune responses through antigen drainage to regional lymph nodes, resulting in activation of circulating immune cells and secondary lymphoid organs, with comparatively limited immune positioning at the respiratory mucosa. (**Right**) (Mucosal BCG vaccination): Localized respiratory delivery directly engages airway epithelial cells and resident innate immune populations, promotes trained innate immunity, and supports the establishment of tissue-resident memory T (T_RM_) cells and donor-unrestricted T-cell populations. These coordinated local immune responses enhance immune positioning at the respiratory interface and may facilitate early containment of *Mycobacterium tuberculosis* (Mtb) following exposure. Overall, the comparison highlights vaccination route as a key determinant of the localization, coordination, and functional organization of protective immune responses.

As summarized in Figure 1, the route of BCG administration determines the spatial organization of vaccine-induced immunity by directing immune responses to distinct anatomical compartments rather than simply amplifying systemic immunity. Compared with parenteral vaccination, mucosal delivery preferentially programs immune responses within the respiratory tract, promoting the establishment of tissue-associated immune compartments, including T_RM_ cells and donor-unrestricted T-cells (DURTs) populations such as γδ T cells and mucosal-associated invariant T (MAIT) cells. These populations are strategically positioned to enable rapid pathogen sensing and coordinate early antimycobacterial immune responses.

Accordingly, the route of vaccination should be regarded as a fundamental design parameter that determines the cellular architecture and spatial organization of protective immunity at the site of Mtb entry. This spatial organization has important implications for how immune responses are initiated, coordinated, and maintained within the respiratory mucosa [18]. Consistent with this concept, human studies have demonstrated that different routes of BCG vaccination generate distinct systemic and mucosal transcriptional programs, supporting the concept of route-dependent immune programming rather than a uniform enhancement of immune responses [8,9].

### 2.1. Historical Emphasis on Parenteral BCG Vaccination

Following its development by Calmette and Guérin, BCG was initially administered via multiple routes, including oral delivery. The first documented human use in 1921 involved oral vaccination of a newborn, reflecting early efforts to mimic the natural route of Mtb exposure and induce protective immunity [2]. Oral BCG vaccination was subsequently implemented in several settings and was considered an attractive strategy for large-scale immunization. However, oral vaccination was gradually abandoned because of variable immunogenicity, inconsistent gastrointestinal uptake of bacilli, difficulties in dose standardization, and safety concerns associated with early vaccine preparations. These limitations ultimately favored parenteral administration—particularly intradermal vaccination—which enabled more standardized and reproducible vaccine delivery and subsequently became the global standard [2,11]. Importantly, this transition was driven primarily by practical considerations rather than by immunological optimization at the site of natural infection. Consequently, research on BCG-induced immunity became largely centered on systemic immune readouts, particularly circulating T-cell responses, thereby implicitly equating protective immunity with systemic cell-mediated immunity.

More recent experimental and clinical studies have challenged this parenteral-centric paradigm by demonstrating that the route of vaccination fundamentally influences the spatial organization of immune responses at mucosal sites. Early intranasal studies showed that mucosal BCG vaccination could enhance protection but also induce pulmonary pathology when immune activation was excessive or dose-dependent [19]. More recently, mechanistic studies have demonstrated that airway epithelial cells actively sense BCG exposure and orchestrate downstream innate immune responses, highlighting that mucosal immunity is governed by tissue-specific regulatory networks rather than representing a simple extension of systemic immunity [20].

Collectively, these findings indicate that the historical predominance of parenteral BCG vaccination has shaped not only experimental approaches but also conceptual models of protective immunity by underrepresenting the contribution of mucosal immune mechanisms.

### 2.2. Mismatch Between the Vaccination Route and the Portal of Infection

A defining feature of TB pathogenesis is that Mtb is typically acquired through the respiratory tract, where the earliest host–pathogen interactions occur within the respiratory mucosa [10]. This tissue comprises a specialized immune environment consisting of epithelial barriers, tissue-resident innate immune cells, and localized lymphoid structures that collectively coordinate early pathogen sensing, immune activation, and pathogen containment. In contrast, conventional BCG vaccination largely bypasses this compartment by inducing immunity predominantly through parenteral administration. This spatial disconnect between the site of immune induction and the portal of pathogen entry raises important questions regarding vaccine efficacy, as systemic priming may not fully engage the mucosal immune networks that govern the earliest stages of infection at the respiratory interface [9,11].

Human studies further underscore this mismatch by demonstrating that parenteral and mucosal BCG vaccination elicit distinct systemic and mucosal transcriptional programs, resulting in qualitative differences in immune organization and function [8,9]. Together, these findings indicate that the route of vaccination determines not only the magnitude of vaccine-induced immunity but also its spatial distribution, tissue localization, and functional organization.

### 2.3. Route-Dependent Qualitative Differences in Immune Responses

Beyond anatomical considerations, the route of BCG vaccination fundamentally influences the quality, localization, and temporal dynamics of vaccine-induced immune responses. Parenteral vaccination predominantly elicits circulating antigen-specific T cells and systemic immune activation, supporting immune surveillance within the blood and secondary lymphoid organs while remaining spatially separated from the primary site of Mtb infection [9,21,22]. In contrast, mucosal delivery redirects immune responses toward the respiratory tract, promoting rapid immune activation within the airways and lung parenchyma. Preclinical studies, including non-human primate models, have demonstrated that alternative routes of BCG administration generate immune profiles that differ not only in tissue localization but also in recall kinetics and functional composition compared with conventional intradermal vaccination [12,13,23]. Consistent with these observations, intranasal BCG vaccination enhances pulmonary protection relative to parenteral administration and is associated with accelerated local immune responses and enhanced antigen-specific interferon (IFN)-γ recall responses, whereas systemic protection remains largely unchanged [24]. Importantly, these route-dependent differences extend beyond adaptive immunity. Mucosal vaccination has been associated with more efficient induction of trained immunity in circulating and bone marrow-derived monocytes than intradermal vaccination, indicating that innate immune reprogramming is also influenced by the site of antigen exposure [23]. Nevertheless, enhanced early immune activation at mucosal sites does not necessarily translate into sustained pulmonary protection. Although mucosal vaccination improves early control of mycobacterial replication within the lung, this advantage may diminish over time, whereas protection against extrapulmonary dissemination appears to be maintained [14,15]. These findings suggest a dissociation between early local containment and long-term disease outcomes. Furthermore, mucosal immune activation requires tight regulation. Intranasal BCG vaccination can enhance protection while simultaneously predisposing the host to dose-dependent pulmonary pathology, highlighting the need to balance protective immunity with the preservation of tissue integrity [19].

Collectively, these observations demonstrate that the route of vaccination reshapes immune architecture across multiple dimensions—including spatial localization, temporal activation, innate immune reprogramming, and tissue-specific regulation—rather than simply augmenting systemic immune responses. Effective protection against TB therefore depends on the coordinated integration of these qualitative features rather than on the magnitude of immune responses alone. The principal route-dependent differences between parenteral and mucosal BCG vaccination are summarized in Table 1.

### 2.4. Engagement of Mucosal Immune Networks Following Localized BCG Delivery

Localized mucosal delivery of BCG directly engages immune networks that are either inaccessible or only indirectly influenced following parenteral vaccination. At the respiratory mucosa, epithelial cells and the surrounding tissue microenvironment contribute to immune programming by producing cytokines and chemokines that coordinate innate and adaptive immune responses [13,20,25]. Airway epithelial cells actively sense mycobacterial components and orchestrate downstream innate immune activation, linking microbial recognition to both pro-inflammatory and regulatory immune pathways [20]. This epithelial-proximal sensing establishes the local tissue context in which subsequent immune responses are initiated and organized. Within the underlying mucosal tissue, dendritic cells (DCs) and alveolar macrophages are exposed to sustained local antigenic stimulation, facilitating antigen presentation, innate immune conditioning, and adaptive priming within the same anatomical compartment. This spatial coupling contrasts with parenteral vaccination, in which antigen encounter, innate immune activation, and T-cell priming occur in anatomically distinct compartments and are separated in both space and time. Mucosal immune tissues further support local antigen recognition, adaptive differentiation, and the establishment of tissue-resident immune populations following mucosal BCG vaccination [13,26]. Together, these processes integrate localized innate activation, adaptive immune programming, and tissue residency more efficiently than parenteral vaccination [13,23,26].

### 2.5. Rationale for Focusing on Mucosal BCG Vaccination

**Figure 2:**
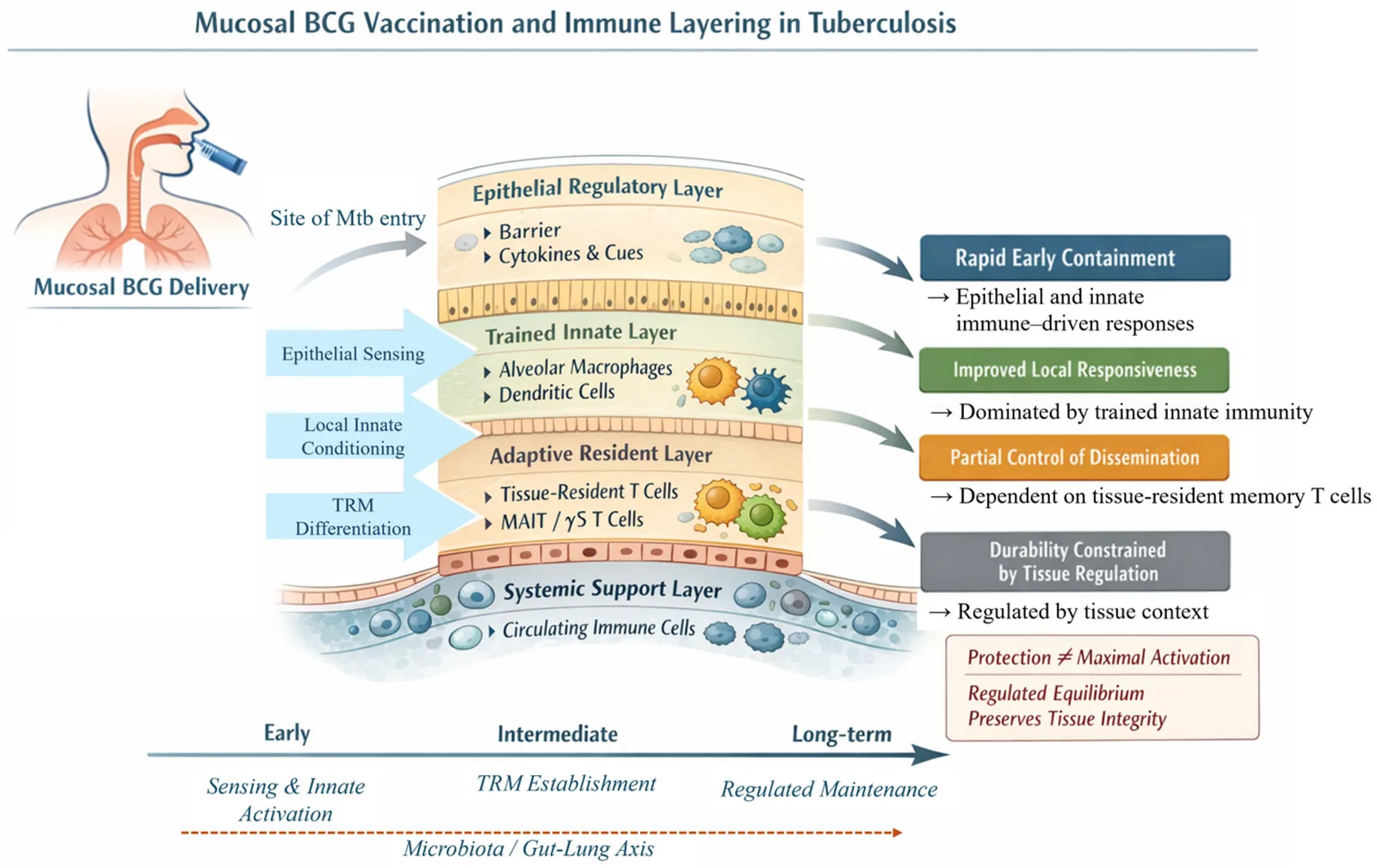
Conceptual framework of immune layering following mucosal BCG vaccination. This schematic illustrates how mucosal BCG vaccination establishes a spatially organized immune architecture at the respiratory interface. Sequential immune layers comprise an epithelial regulatory layer, a trained innate layer, an adaptive resident layer, and a systemic support layer that together coordinate local immune responses. The temporal progression from epithelial sensing and innate activation to tissue-resident memory T (T_RM_) cell establishment and long-term immune maintenance is shown schematically. Functional outcomes include rapid early containment, enhanced local responsiveness, partial control of mycobacterial dissemination, and durability constrained by tissue-specific regulatory mechanisms. Microbiota-derived signals and the gut–lung axis are proposed to influence multiple immune layers over time. The layers represent interacting functional compartments rather than discrete anatomical structures.

Collectively, these observations establish the route of vaccination as a fundamental determinant of immune programming rather than merely a technical variable. The historical reliance on parenteral BCG vaccination has focused immunological evaluation primarily on systemic immune readouts, providing comparatively limited insight into the immune mechanisms operating at the respiratory mucosa [8,9,11]. Renewed interest in mucosal BCG vaccination reflects increasing recognition of these limitations. By aligning immune induction with the anatomical site of natural infection, mucosal delivery reveals immunological features that are not fully captured by systemic measurements alone, including localized trained innate immunity, tissue-resident memory formation, and coordinated immune responses within the respiratory tract [11,12,23,26]. Importantly, mucosal immune programming is a tightly regulated process rather than a simple amplification of immune activation. The balance between protective immunity and the preservation of tissue integrity, governed by epithelial sensing, innate immune conditioning, and adaptive differentiation, is a key determinant of vaccine outcome [19,20]. Within this framework, mucosal BCG vaccination provides a model of immune organization in which the route of vaccination functions as a primary design parameter governing the spatial organization, temporal dynamics, and functional specialization of vaccine-induced immunity. This concept of “immune layering,” summarized in Figure 2, provides the mechanistic foundation for the subsequent sections of this review.

### 2.6. Distinct Mucosal Delivery Routes and Their Effects on Immune Layering

Although mucosal BCG vaccination is frequently discussed as a single immunological category, distinct mucosal delivery routes engage the immune-layering framework in different ways. Intranasal and intratracheal administration directly target the respiratory mucosa and efficiently promote epithelial conditioning, trained innate immunity, and T_RM_ cell formation within the lung. Aerosol delivery may provide broader coverage of the respiratory tract while retaining many features of local immune programming. In contrast, oral BCG vaccination primarily engages gastrointestinal immune networks and may influence respiratory immunity indirectly through microbiota-dependent mechanisms operating along the gut–lung axis. These observations suggest that individual mucosal routes differentially shape each immune layer and should therefore be considered distinct immunological strategies rather than interchangeable delivery methods. A comparative overview of their anticipated effects on epithelial regulation, trained innate immunity, tissue-resident adaptive memory, and systemic immune support is provided in Table 2.

## 3. Coordinated Innate and Adaptive Immune Programming by Mucosal BCG Vaccination

Building on the spatial framework outlined in Section 2, the route of BCG administration determines not only where immune responses are localized but also how innate and adaptive immune components are coordinated over time. Rather than functioning independently, these compartments form an integrated immune network in which early innate sensing shapes downstream adaptive differentiation, including DURTs programs enriched at mucosal sites [18,27]. A central component of this coordination is trained immunity, a form of innate immune memory mediated by epigenetic and metabolic reprogramming of innate immune cells. Although initially characterized as a systemic consequence of parenteral BCG vaccination, trained immunity is now recognized as a spatially organized process that can also be established locally within tissue-resident innate immune populations. Consequently, the site of BCG exposure determines whether innate immune programming is distributed predominantly through systemic compartments or reinforced locally within barrier tissues such as the respiratory mucosa. At mucosal sites, trained innate immunity influences subsequent adaptive immune responses by modulating antigen presentation, cytokine availability, and the local tissue environment in which T-cell priming and differentiation occur. Consistent with this concept, mucosal BCG vaccination has been associated with more efficient induction of trained immunity in circulating and bone marrow-derived monocytes than intradermal vaccination [23].

The consequences of this coordinated immune organization are particularly evident in its temporal dynamics. Mucosal BCG vaccination promotes rapid immune activation within the lung, establishing localized innate immune training that facilitates the development of tissue-associated adaptive immune responses [13,14,23]. However, this early advantage does not consistently translate into durable pulmonary protection, whereas protection against extrapulmonary dissemination and improved survival may persist [14,15]. These observations indicate that early local containment and long-term disease outcomes are only partially coupled. Within this framework, trained innate immunity represents the initial layer of immune organization that conditions—but does not fully determine—subsequent adaptive immune responses. This interpretation is further supported by human studies showing that BCG-induced immune reprogramming does not uniformly confer durable protection, highlighting the importance of downstream regulatory mechanisms and tissue-specific contexts in shaping long-term immunity [17,27].

### 3.1. Trained Innate Immunity at Mucosal Sites

A key consequence of mucosal BCG vaccination is the induction of trained immunity, a form of innate immune memory mediated by epigenetic and metabolic reprogramming of innate immune cells within barrier tissues [3,4,5,6,7]. Although initially described in circulating innate immune cells following parenteral vaccination, trained immunity can also be established locally within tissue-resident innate immune populations at mucosal sites. Among these populations, alveolar macrophages and DCs serve as the principal mediators of mucosal trained immunity, acquiring persistent tissue-adapted phenotypes shaped by local environmental signals [28,29,30]. Mucosal BCG delivery directly exposes these cells to mycobacterial stimuli, promoting spatially localized and sustained innate immune reprogramming. This process is driven by coordinated epigenetic remodeling and metabolic rewiring involving signaling pathways such as mechanistic target of rapamycin (mTOR) and hypoxia-inducible factor (HIF)-1α [5,6,31,32,33], thereby enhancing cytokine production and strengthening early antimicrobial responses. Trained immunity at mucosal sites may also arise indirectly. Parenteral BCG vaccination has been shown to induce lung-resident trained macrophages through microbiota-dependent signaling along the gut–lung axis [34], indicating that systemic vaccination can influence mucosal innate immunity through distal regulatory mechanisms. The magnitude and persistence of trained immunity are further influenced by host-derived and microbiota-derived signals, including epithelial mediators and microbial metabolites, which shape myeloid cell responsiveness and may contribute to inter-individual variability in vaccine responses [25,35]. In addition, recombinant BCG strains have demonstrated the capacity to enhance local innate immune activation, suggesting that mucosal innate programming can be further optimized through vaccine engineering [36].

Recent studies have further refined our understanding of tissue-resident myeloid populations within the trained innate layer. Emerging evidence suggests that alveolar macrophages not only acquire durable trained phenotypes but also function as long-lived tissue organizers that shape local immune responsiveness following vaccination [28,37]. In parallel, dendritic cell populations contribute to the integration of innate and adaptive immunity by translating local innate activation into tissue-specific T-cell differentiation and functional specialization programs [30,38]. These findings support the concept that alveolar macrophages and dendritic cells serve as key cellular interfaces linking epithelial sensing, trained innate immunity, and adaptive tissue residency, thereby reinforcing the mechanistic basis of immune layering at the respiratory mucosa. Together, these studies extend the trained innate layer beyond the concept of enhanced cytokine responsiveness and support a broader role for tissue-resident myeloid populations as coordinators of cross-layer communication within the immune-layering framework [27,30,37,38].

Collectively, these findings support a model in which BCG establishes trained innate immunity at mucosal sites through complementary local and systemic pathways. This trained innate compartment represents the initial layer of immune organization, linking the route of vaccination to the broader framework of immune layering.

### 3.2. Tissue-Resident Memory T Cells as an Adaptive Component of Immune Layering

T_RM_ cells constitute the principal adaptive resident layer induced by mucosal BCG vaccination. In this section, we emphasize their position within the broader immune-layering framework rather than repeating the detailed mechanisms of T_RM_ differentiation and maintenance, which are discussed in Section 4. Unlike circulating memory T cells, T_RM_ cells are strategically positioned within non-lymphoid tissues and can provide rapid local responses at the site of pathogen entry [21]. Mucosal BCG vaccination preferentially enriches antigen-specific lung T_RM_ populations compared with parenteral vaccination, including programmed cell death protein 1 (PD-1)+ killer cell lectin-like receptor G1 (KLRG1)− CD4^+^ T_RM_ cells associated with improved early pulmonary control [39]. These findings support the role of T_RM_ cells as a rapid-response adaptive layer that complements trained innate immunity during the earliest stages of Mtb exposure.

However, TRM-mediated early containment should not be equated with durable sterilizing immunity. Although mucosal BCG vaccination enhances early pulmonary protection, whether these early local responses translate into sustained long-term protection remains unclear [14,17,39]. This suggests that durable protection likely depends on the coordinated contribution of multiple immune layers rather than TRM cells alone. Accordingly, T_RM_ cells are best viewed as one adaptive component within an integrated immune network that also requires epithelial regulation, trained innate conditioning, systemic immune support, and tissue-specific homeostatic constraints. This framing reduces overlap with Section 4 and clarifies that the biological mechanisms governing T_RM_ generation, persistence, and vaccine redesign are discussed in greater detail in the following section.

### 3.3. Interface Between Trained Innate Immunity and Adaptive Residency

Rather than restating the full differentiation pathway of T_RM_ cells, this section highlights how trained innate immunity creates the conditions that favor adaptive residency. Following mucosal BCG delivery, tissue-resident macrophages and DCs are exposed to local mycobacterial stimuli and epithelial-derived cues, leading to epigenetic and metabolic reprogramming that alters antigen presentation, cytokine availability, and the local inflammatory milieu [28,29,30,31,32,33]. These trained innate populations thereby condition the microenvironment in which antigen-specific T cells are recruited, retained, and functionally specialized. In this sense, trained immunity serves as an upstream organizing layer that helps determine whether adaptive responses remain circulating or become embedded within the respiratory tissue.

This interface between innate conditioning and adaptive residency provides the mechanistic bridge between Section 3 and Section 4. Section 3 defines the coordinated immune architecture induced by mucosal BCG vaccination, whereas Section 4 examines the specific signals, tissue niches, and regulatory constraints that govern T_RM_ differentiation and persistence in greater detail.

### 3.4. Temporal Coordination of Early Local Containment and Long-Term Protection

The protective effects of mucosal BCG vaccination unfold over time and depend on the coordinated activity of multiple immune layers rather than on the persistence of a single effector population. Early after vaccination or challenge, epithelial sensing, trained innate responsiveness, and T_RM_-mediated recall responses act together to accelerate pulmonary immune activation and improve early containment of Mtb [13,14,20,26]. Over longer intervals, however, this early advantage may be limited by tissue-specific regulatory mechanisms, changing antigen availability, and the capacity of local niches to sustain resident immune populations without compromising pulmonary homeostasis [19,40,41,42]. Thus, the temporal dissociation between early pulmonary control and durable protection reflects a central feature of immune layering.

This temporal perspective further clarifies the distinction between Section 3 and Section 4. Section 3 addresses how immune layers are coordinated as a system, whereas Section 4 focuses on the cellular and tissue-level determinants that make T_RM_ responses durable, functional, and compatible with mucosal homeostasis.

### 3.5. Molecular and Cellular Communication Across Immune Layers

The concept of immune layering implies that protective immunity arises not simply from the coexistence of distinct immune compartments but from continuous communication among them. Although many of these interactions remain incompletely defined, current evidence supports the existence of several molecular and cellular pathways linking the epithelial regulatory, trained innate, adaptive resident, and systemic support layers. At the respiratory interface, epithelial cells act as the initial sensing layer and respond to BCG exposure through the production of cytokines, chemokines, and growth factors. These mediators influence the activation state of alveolar macrophages and dendritic cells, thereby linking epithelial sensing to trained innate immune programming. In particular, epithelial-derived cytokines, including members of the interleukin (IL)-1 family, granulocyte-macrophage colony-stimulating factor (GM-CSF), and transforming growth factor-β (TGF-β), contribute to the local immune environment in which innate and adaptive immune responses are initiated and regulated [20,25,38,40]. Communication between the trained innate and adaptive resident layers is mediated primarily through antigen-presenting cells. Trained macrophages and dendritic cells exhibit enhanced cytokine production, antigen presentation, and responsiveness to secondary stimulation, thereby influencing T-cell activation and tissue residency programs. These functions shape the local conditions that promote the generation and maintenance of tissue-resident memory T cells following mucosal vaccination [28,29,31,32,38,40,43]. Bidirectional communication is also evident once T_RM_ cell populations are established. Upon pathogen encounter, T_RM_ cells rapidly produce effector cytokines such as IFN-γ and TNF, which enhance the antimicrobial activity of local innate immune cells and reinforce protective responses at the site of infection [21,39]. Thus, trained innate immunity and tissue-resident adaptive memory operate as a functional circuit rather than as independent compartments. A further level of communication is provided by microbiota-derived metabolites and systemic signals transmitted through the gut–lung axis. These factors can simultaneously influence epithelial function, innate immune metabolism, and adaptive immune differentiation, thereby coordinating multiple immune layers across tissues. Together, these observations support a model in which immune layering is maintained through interconnected cytokine networks, antigen-presenting cell conditioning, metabolic programming, and microbiota-dependent regulation. Although many of these pathways remain to be validated experimentally, they provide a mechanistic basis for the coordinated immune organization induced by mucosal BCG vaccination.

### 3.6. Coordinated Immune Layering as a Framework for BCG-Induced Protection

The findings discussed above indicate that the immunological effects of BCG vaccination cannot be fully understood by considering innate and adaptive immunity as independent processes. Rather, mucosal BCG vaccination promotes coordinated interactions between trained innate immunity and adaptive immune responses through route-dependent immune programming [23,27,30,43]. Within this framework, protection against pulmonary TB depends not only on the magnitude of vaccine-induced immune responses but also on their spatial organization, temporal coordination, and functional specialization. Trained innate immunity shapes the local tissue environment and supports downstream adaptive responses, including the induction of T_RM_ cells, thereby establishing an integrated immune network at the respiratory mucosa [11,13,23,26,28,43]. This perspective also helps explain the variable efficacy of BCG vaccination. Differences in protection may reflect the efficiency with which immune layering is established, coordinated, and maintained within mucosal tissues rather than differences in vaccine formulation or host characteristics alone. Consistent with this concept, human studies demonstrate that BCG-induced immune responses do not uniformly translate into protection against Mtb infection, underscoring the importance of tissue-specific regulation and immune integration in determining vaccine efficacy [17,27]. The implications of this framework extend beyond TB. Vaccination strategies capable of simultaneously inducing trained innate immunity, tissue-resident adaptive immunity, and appropriately regulated tissue responses may provide broader protection against pathogens that enter through mucosal surfaces [13,31]. Likewise, experimental approaches that achieve robust protection further illustrate the profound influence of the vaccination route on immune organization. In particular, intravenous BCG vaccination induces extensive pulmonary immune seeding and remarkable protection against Mtb challenge in non-human primates, although its clinical applicability remains limited [44,45]. Protection is associated with airway-localized adaptive responses, including polyfunctional CD4+ T cells, Th1/Th17-associated populations, natural killer (NK) cells, and mycobacteria-responsive γδ T cells associated with protection [44,45,46], as well as early innate transcriptional programs involving type I interferon signaling and dendritic-cell activation [47,48]. More recent studies further demonstrate that intravenous BCG establishes a coordinated pulmonary immune ecosystem characterized by activated alveolar macrophages, enhanced myeloid–T-cell communication, and humoral correlates of protection, including antigen-specific IgM, complement-associated signatures, and NK-cell-activating antibodies [45,48,49]. Within the immune-layering framework, these findings suggest that intravenous vaccination establishes multiple immune layers through systemic dissemination and pulmonary immune compartmentalization rather than direct mucosal instruction. Consequently, immune layering may be viewed as a continuum of route-dependent immune organization rather than a simple dichotomy between parenteral and mucosal vaccination.

Together, these observations support coordinated immune layering as a unifying conceptual framework linking the mechanisms of mucosal immunity with vaccine performance. Rather than being determined solely by the magnitude of early immune activation, durable protection emerges from the dynamic integration of epithelial regulation, trained innate immunity, tissue-resident adaptive memory, systemic immune support, and tissue-specific regulatory processes over time.

To make the immune-layering framework experimentally tractable, each layer should be defined not only conceptually but also through measurable candidate biomarkers and operational readouts. These markers should be interpreted as provisional criteria rather than established correlates of protection because their relative contributions to durable immunity remain to be validated across preclinical models and clinical studies. Nevertheless, defining such readouts provides a practical basis for testing whether mucosal BCG vaccination establishes coordinated epithelial, innate, adaptive resident, and systemic immune programs. A summary of the proposed operational definitions, representative biomarkers, and experimental and clinical assessment strategies for each immune layer is provided in Table 3.

This operational framework emphasizes that immune layering should be evaluated through integrated, tissue-informed endpoints rather than through single systemic correlates alone. In particular, the most informative studies will likely combine local airway or tissue sampling with peripheral immune profiling and longitudinal protection outcomes, thereby determining whether individual biomarkers represent causal contributors to protection, surrogate markers of immune organization, or context-dependent indicators of tissue compatibility.

**Figure 3:**
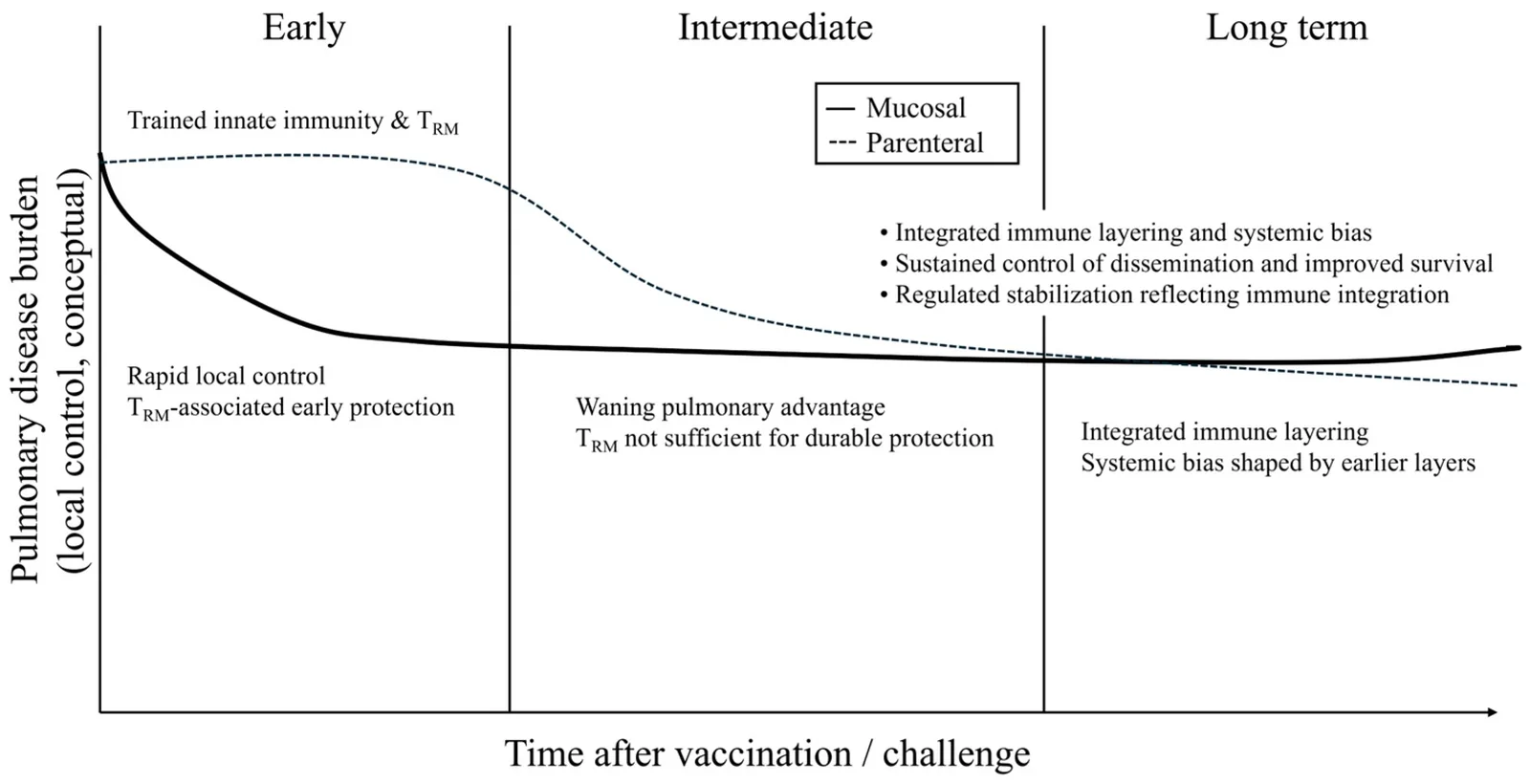
Temporal dynamics of route-dependent protection following BCG vaccination. Conceptual model illustrating the temporal evolution of immune protection following mucosal and parenteral BCG vaccination. Mucosal vaccination promotes rapid early control of *Mycobacterium tuberculosis* (Mtb) through coordinated epithelial sensing, trained innate immunity, and tissue-resident memory T (T_RM_) cell responses, resulting in enhanced early pulmonary protection. This initial advantage gradually diminishes as T_RM_-mediated immunity alone becomes insufficient to sustain durable pulmonary protection, leading to convergence of protection between vaccination routes during the intermediate phase. At later stages, protective immunity is increasingly determined by the coordinated integration of multiple immune layers—including innate, adaptive, systemic, and regulatory components—rather than by early local responses alone. The model emphasizes that durable protection emerges from dynamic immune integration over time rather than from the magnitude of the initial immune response.

Viewed through this operational framework, the evolving relationship between early local containment and long-term protection can be interpreted as the outcome of dynamic interactions among multiple immune layers. The temporal progression of these interactions and their contribution to durable protection are illustrated conceptually in Figure 3.

## 4. Formation and Maintenance of Tissue-Resident Memory T Cells in Mucosal Tissues: T_RM_ as a Key Target for BCG Redesign

Section 3 established T_RM_ cells as the adaptive resident component of mucosal BCG-induced immune layering. Building on this framework, the present section focuses on the mechanisms governing T_RM_ cell generation, maintenance, and functional specialization, as well as their implications for rational BCG redesign. Although lung-resident T_RM_ populations contribute substantially to the early containment of Mtb at the respiratory interface [21,39], durable protection depends on whether these cells are generated within the appropriate anatomical context, supported by compatible tissue niches, and regulated such that local immune readiness does not progress to immunopathology.

Accordingly, Section 4 is organized around T_RM_-specific vaccine design questions: which signals drive tissue residency, which tissue niches sustain long-term persistence, which regulatory constraints limit protective durability, and how BCG-based strategies might be engineered to optimize these processes. This organization distinguishes the mechanistic treatment of T_RM_ biology from the systems-level immune-layering framework presented in Section 3.

### 4.1. Signals Governing T_RM_ Differentiation at Mucosal Sites

The differentiation of T_RM_ cells at mucosal sites is a context-dependent process governed by local tissue signals and the anatomical site of immune activation. During mucosal BCG vaccination, these factors determine whether activated T cells adopt circulating memory phenotypes or establish stable tissue residency [13,26,43,50]. A key determinant is the anatomical context of immune priming. T cells activated within mucosal tissues encounter antigen-presenting cells (APCs) conditioned by epithelial and stromal signals, promoting tissue retention over systemic recirculation and facilitating the establishment of lung-resident memory populations [11,13,39,43]. This differentiation program is further shaped by local cytokine and epithelial-derived cues, including TGF-β-associated pathways, which promote both the establishment and long-term maintenance of tissue residency [21,39,43]. In parallel, coordinated metabolic and epigenetic programming generates durable tissue-adapted memory states that support long-term persistence within the mucosal environment [42,50,51]. Prolonged antigen presentation following mucosal BCG vaccination may extend interactions between T cells and locally conditioned APCs, thereby promoting tissue-residency programs and TRM differentiation within the lung microenvironment [13,38,43].

Together, these observations indicate that T_RM_ differentiation is governed by the integration of anatomical priming, local immune conditioning, metabolic–epigenetic programming, and antigen persistence. From a vaccine design perspective, these complementary mechanisms represent key leverage points for enhancing tissue-resident memory through optimized BCG vaccination strategies.

### 4.2. Tissue Niches Supporting Long-Term T_RM_ Maintenance

The long-term protective capacity of T_RM_ cells depends not only on their differentiation but also on their sustained maintenance within specialized tissue niches. Building on the differentiation signals discussed in Section 4.1, the persistence of T_RM_ cells depends on their ability to establish and maintain interactions with supportive tissue microenvironments. Unlike circulating memory T cells, T_RM_ cells occupy spatially restricted niches that provide the structural, cytokine, and metabolic support required for long-term survival and functional readiness [21,42,50]. These niches are maintained through coordinated interactions among tissue-resident innate immune cells and non-hematopoietic structural components. Resident macrophages contribute to local immune homeostasis by regulating cytokine availability and thereby indirectly supporting T_RM_ cell persistence [40]. In parallel, epithelial and stromal cells shape tissue architecture while providing retention and survival signals that sustain tissue-resident immune memory [40,43,50]. Metabolic adaptation represents another defining feature of T_RM_ cell niches. Local tissue environments impose physiological constraints, including limited nutrient availability and fluctuating oxygen tension, to which T_RM_ cells adapt through specialized metabolic programs that support long-term survival and functional competence [42,50]. Importantly, these niches are dynamic rather than static. Tissue architecture, stromal composition, and metabolic capacity continually adapt in response to environmental exposure and immune activation and are further influenced by route-dependent innate conditioning, including trained immunity [23,40,42]. Consequently, T_RM_ cell maintenance reflects a regulated equilibrium sustained through continuous interactions with a changing tissue microenvironment. From a vaccine design perspective, durable tissue-resident immunity requires not only efficient induction of T_RM_ cells but also the successful establishment, stabilization, and long-term maintenance of supportive tissue niches. Accordingly, the quality of T_RM_-supportive microenvironments emerges as a critical determinant of long-term protection at mucosal surfaces.

### 4.3. Environmental and Regulatory Constraints on T_RM_ Persistence

Although supportive tissue niches are essential for T_RM_ cell maintenance, their persistence is ultimately constrained by the physiological requirements of tissue homeostasis. At mucosal surfaces, immune memory must remain compatible with continuous environmental exposure, tissue remodeling, and regulatory mechanisms that prevent excessive inflammation. Consequently, T_RM_ cell persistence is best viewed as a dynamically regulated equilibrium rather than a fixed endpoint [50]. One of the principal constraints arises from the inflammatory environment. Although transient inflammation promotes T_RM_ cell establishment, sustained or excessive immune activation can compromise tissue integrity, destabilize supportive niches, and impair T_RM_ cell function. This balance is particularly important in the lung, where immune responses must preserve efficient gas exchange. Excessive immune activation may therefore promote immunopathology rather than enhance protection [19,29,42]. Consistent with this concept, intranasal BCG vaccination enhances pulmonary protection but may also induce dose-dependent granulomatous pathology [19]. Conversely, insufficient immune stimulation may limit _TRM_ cell persistence because tightly regulated mucosal environments can restrict activation thresholds, survival signals, and the effector readiness of resident memory populations [21,42,50]. Additional constraints arise from tissue-specific adaptation and progressive changes within the local microenvironment. T_RM_ cell populations exhibit substantial functional heterogeneity across tissues, whereas age-associated remodeling of stromal and metabolic niches may progressively impair their maintenance [42,50]. Importantly, persistent T_RM_ cell responses are not universally beneficial. Excessive or dysregulated persistence of resident immune populations may contribute to chronic inflammation and tissue injury, emphasizing that durable protective immunity requires compatibility with tissue-specific regulatory mechanisms rather than simply maximizing tissue-resident memory [29,52].

Together, these observations demonstrate that long-term T_RM_ cell persistence depends not only on supportive tissue niches but also on their compatibility with dynamic environmental and regulatory constraints. From a vaccine design perspective, strategies should therefore optimize the induction and maintenance of tissue-resident memory while preserving tissue integrity and immune homeostasis, rather than simply maximizing T_RM_ cell abundance.

### 4.4. Implications for BCG Redesign: Engineering Tissue-Resident Memory T Cell Responses

The mechanisms discussed in Section 4.1, Section 4.2 and Section 4.3 indicate that T_RM_ cells represent programmable immune states that can be deliberately shaped through rational vaccine design. Accordingly, optimizing the generation, maintenance, and function of T_RM_ cells has emerged as a central objective for improving BCG-based vaccination strategies against pulmonary TB. A primary design parameter is the route of antigen delivery, which determines the spatial and temporal context of immune priming. Mucosal vaccination aligns antigen presentation, innate immune conditioning, and tissue-specific imprinting within the respiratory tract, thereby promoting T_RM_ cell differentiation and establishing localized immune memory. In contrast, parenteral vaccination primarily engages systemic immune compartments and is less effective at generating the tissue microenvironments that support durable T_RM_ cell development. Consistent with this concept, intranasal and intratracheal BCG vaccination in experimental models induces antigen-specific T_RM_ cell populations within the lungs and airways and confers superior pulmonary protection compared with conventional parenteral vaccination [11,13,26,53]. Successful T_RM_-oriented vaccine design also requires precise regulation of innate immune conditioning. Although trained innate immunity promotes adaptive memory formation, its benefits depend on achieving a balanced activation state that remains compatible with tissue homeostasis. Excessive or dysregulated innate activation may compromise supportive tissue microenvironments, whereas insufficient conditioning may limit the establishment of effective tissue-resident immunity. These observations emphasize the importance of calibrated and sustained innate immune programming [23,28,43]. Antigen dynamics constitute an additional determinant of T_RM_ cell development. Prolonged antigen presentation following mucosal BCG vaccination may extend interactions between T cells and locally conditioned APCs, thereby promoting tissue-residency programs within the lung microenvironment [13,38,43]. Human studies further demonstrate that immune reprogramming alone does not necessarily confer sustained protection against Mtb infection [17], indicating that successful vaccine design must balance durable immune memory with preservation of tissue integrity and regulatory homeostasis [19,29,42].

Together, these findings define several complementary principles for T_RM_-centered vaccine optimization, including route-dependent immune priming, calibrated innate immune conditioning, controlled antigen persistence, and compatibility with tissue-specific regulatory mechanisms. Within this framework, mucosal BCG vaccination provides a blueprint for translating the concept of immune layering into rational vaccine design, highlighting the need for tissue-informed immunological endpoints that capture localized, functionally specialized, and durable protective immunity.

## 5. Regulatory Layers Shaping Mucosal BCG Immunity: Roles of Epithelial Cells and the Resident Microbiota

### 5.1. Mucosal Immune Regulation as a Distinct Layer of BCG-Induced Immunity

Immune responses elicited by BCG vaccination have traditionally been described in terms of innate activation and adaptive priming. As discussed in the preceding sections, however, their induction, spatial organization, and long-term persistence are fundamentally influenced by regulatory mechanisms operating at mucosal surfaces. Within barrier tissues, immune activation must be balanced against continuous environmental exposure while preserving tissue integrity, necessitating regulatory networks that shape both the magnitude and quality of immune responses. These mucosal regulatory systems function as active, instructive networks that determine local immune tone, cellular differentiation, and tissue organization. Through epithelial-derived mediators, microbiota-derived metabolites, and tissue-specific signaling pathways, they influence whether immune activation results in protective immunity, immune tolerance, or immunopathology [25,54]. Importantly, this regulation is inherently spatial. Immune responses are compartmentalized within localized tissue niches in which activation thresholds, cellular interactions, and effector functions are continuously modulated. Such spatial regulation promotes T_RM_ cell establishment while limiting excessive inflammation and preserving tissue integrity [21,50].

Mucosal regulatory networks also influence trained innate immunity. Epithelial sensing and microbiota-derived signals calibrate innate immune activation toward protective rather than excessive inflammatory responses [20,29,52]. These regulatory pathways further contribute to inter-individual variability in vaccine responsiveness [25,35,54]. Moreover, mucosal immune regulation is dynamic, evolving in response to environmental exposure and changes in microbial community composition. Consequently, vaccine-induced protection is best viewed as a context-dependent equilibrium rather than a fixed immunological outcome [54,55].

Together, these principles establish mucosal immune regulation as a fundamental layer of BCG-induced immunity that integrates spatial, environmental, and metabolic signals to shape protective immune responses. This regulatory framework provides the foundation for the epithelial- and microbiota-mediated mechanisms discussed in the following sections.

### 5.2. Epithelial-Centered Immunoregulatory Circuits at Mucosal Surfaces

At mucosal surfaces, epithelial cells function as active regulators of immune architecture rather than passive physical barriers [25,55,56]. Positioned at the interface between the external environment and host tissues, they integrate microbial and environmental signals and translate them into spatially localized cues that establish the context for immune activation [55,57]. Consistent with this role, innate immune activation initiated at the respiratory mucosa precedes downstream adaptive responses and can influence the outcome of mycobacterial infection [20,38]. A defining feature of epithelial regulation is its instructional capacity. Through the controlled release of cytokines, chemokines, and other soluble mediators, epithelial cells regulate the activation state of tissue-resident myeloid cells while maintaining local immune homeostasis [55,56,57,58]. This epithelial conditioning also directs adaptive immune responses. APCs operating within epithelial microenvironments acquire properties that support T_RM_ cell differentiation, including through TGF-β-dependent pathways arising from epithelial–immune crosstalk [22,43,59]. Experimental studies further demonstrate that epithelial sensing of mycobacterial exposure precedes and orchestrates innate immune activation through the induction of early cytokine and chemokine responses [20].

Together, these observations position epithelial regulatory circuits as a central layer of mucosal immunity that links innate immune conditioning with adaptive memory formation while coordinating protective immune responses with the preservation of tissue integrity.

### 5.3. Resident Microbiota as Modulators of Mucosal BCG Responses

The resident microbiota modulates mucosal immune responses to BCG by shaping epithelial regulatory programs while also exerting direct effects on immune cell function [35,54,55]. Through continuous interactions with epithelial cells, commensal microorganisms influence epithelial physiology and immunoregulatory activity, thereby conditioning the tissue environment in which vaccine-induced immune responses develop. Microbiota-derived metabolites regulate epithelial gene expression and cytokine production, modulating immune responsiveness and indirectly influencing myeloid cell function while contributing to inter-individual variability in vaccine responses [25,35,54]. The microbiota–host axis also regulates trained innate immunity. By influencing host metabolism and local cytokine environments, commensal microbial communities determine both the magnitude and functional quality of trained immune responses, resulting in context-dependent immune modulation rather than uniform activation [23,25,35,54].

Together, the resident microbiota functions as a critical regulator of immune calibration, integrating microbial and metabolic signals into epithelial-centered regulatory circuits that determine whether vaccine-induced immune reprogramming is translated into durable protective immunity.

### 5.4. The Gut–Lung Axis as a Systemic Regulatory Circuit

The gut–lung axis represents a systemic regulatory network through which microbiota-derived signals originating in the intestine influence immune responsiveness at distal mucosal sites, including the respiratory tract. These effects are mediated by circulating microbial metabolites and other bioactive products that calibrate innate and adaptive immune responses within the lung. Disruption of the intestinal microbiota, for example through antibiotic treatment, impairs immune function at the respiratory mucosa, underscoring the functional interdependence between intestinal and pulmonary immunity [54,60,61]. Accordingly, the gastrointestinal and respiratory tracts are linked by systemic regulatory pathways that continuously recalibrate mucosal immune tone across tissues [54,60,62]. Gut microbiota-derived metabolites enter the circulation and influence systemic innate immune cells by modifying their metabolic and epigenetic states, thereby altering their capacity for trained innate immune reprogramming [35,52,54]. These systemic signals establish the regulatory context in which local epithelial and myeloid immune responses are initiated. Importantly, BCG vaccination engages this regulatory circuit even after parenteral administration. By altering gut microbiota composition and associated systemic signaling, BCG promotes trained innate immunity in lung-resident macrophages, illustrating how distinct vaccination routes may converge on shared regulatory pathways [34,35,63]. The gut–lung axis also influences the inflammatory and metabolic landscape of the respiratory tract, thereby affecting adaptive immune compartmentalization and biasing immune responses toward tissue residency or systemic recirculation [54,60,61].

Together, these findings establish the gut–lung axis as a systemic regulatory layer that integrates distal microbial signals with local immune programming, thereby coordinating immune activation with tissue-specific regulatory capacity.

### 5.5. Balancing Protection and Homeostasis: Regulatory Constraints on Mucosal BCG Immunity

Protective immunity at mucosal surfaces is fundamentally constrained by the need to preserve tissue homeostasis. At barrier sites, immune responses must remain compatible with continuous environmental exposure and normal physiological function, such that protection is achieved through a dynamically regulated immune equilibrium rather than maximal immune activation [29,52,55]. Although trained innate immunity enhances resistance to secondary infection, excessive or dysregulated activation may promote inflammatory pathology [19,29,52]. These responses are regulated in part by epithelial-derived mediators and microbiota-associated signals that maintain local immune homeostasis [25,52,55]. Likewise, T_RM_ cells provide rapid localized protection, but their differentiation, maintenance, and function are tightly controlled by tissue-specific regulatory mechanisms to ensure effective immunity while minimizing tissue damage [21,50]. Systemic regulatory inputs mediated through the gut–lung axis further influence mucosal immune tone following vaccination, whereas temporal changes in microbiota composition may contribute to inter-individual variability in vaccine responsiveness [35,60,62]. Importantly, clinical studies demonstrate that BCG-induced immune responses do not consistently translate into sustained protection against Mtb infection [17].

Together, these observations indicate that durable vaccine efficacy depends on the successful integration of protective immune responses with tissue-specific regulatory mechanisms that preserve pulmonary homeostasis.

### 5.6. Integration of Epithelial and Microbial Regulation into Immune Layering

Mucosal immunity induced by BCG vaccination emerges through the coordinated integration of regulatory, innate, and adaptive immune processes rather than through the independent activation of individual immune compartments. Within this framework, epithelial barrier systems establish the local regulatory context by integrating environmental and microbial signals and translating them into tissue-specific programs that shape immune activation [55,57]. The resident microbiota further refines these regulatory processes through microbial metabolites and signaling pathways that influence cytokine production, epithelial function, and immune cell activity [25,54]. Within this coordinated environment, trained innate immunity conditions downstream adaptive differentiation, while T_RM_ cells provide localized protection at the respiratory interface. Tissue residency therefore reflects continuous regulation by local microenvironmental cues rather than antigen exposure alone [28,42,50]. Systemic regulatory circuits, particularly the gut–lung axis, extend these interactions beyond individual tissues, enabling distinct vaccination routes to converge on shared regulatory architectures that shape mucosal immune responses [34,60].

Together, these interconnected regulatory, innate, and adaptive layers constitute a unified framework of immune layering in which durable protection against pulmonary TB emerges from coordinated immune organization rather than isolated immune activation. This perspective provides a conceptual foundation for the rational design of next-generation mucosal vaccines that maximize protective efficacy while preserving tissue-specific immune homeostasis.

## 6. Implications for Vaccine Design and Clinical Translation: Toward Next-Generation Mucosal BCG-Based Vaccines

The immunological principles emerging from mucosal BCG vaccination challenge reductionist views of vaccine efficacy and extend beyond TB. Durable mucosal protection arises not from maximal activation of a single immune component but from the coordinated integration of epithelial, regulatory, innate, and adaptive immune layers within tissue-specific contexts. This framework has important implications for the rational design, evaluation, and clinical translation of next-generation BCG-based vaccines. A central implication is that the route and context of vaccine delivery should be regarded as primary design parameters rather than logistical considerations. Mucosal vaccination establishes the spatial and temporal framework for immune priming, thereby influencing epithelial instruction, microbiota-dependent regulation, trained innate immunity, and T_RM_ cell formation. Consistent with this concept, human studies demonstrate that different routes of BCG vaccination generate qualitatively distinct immune programs rather than simply amplifying a common response [8,9]. Mucosal delivery therefore enables the deliberate programming of immune layering at barrier tissues aligned with the natural portal of Mtb entry.

Within this framework, successful vaccine design cannot be equated with maximal immune stimulation. Because mucosal tissues impose regulatory constraints to preserve barrier integrity and physiological function, excessive or poorly controlled inflammation may destabilize tissue microenvironments and compromise long-term protection. Accordingly, the principal objective of vaccine design is to achieve calibrated immune programming that balances protective immunity with tissue homeostasis.

### 6.1. Reframing Non-Replicating BCG: EFD-BCG as a Regulatory Scaffold

The immune-layering framework also provides a basis for re-evaluating non-replicating BCG formulations. Historically, the limited efficacy of killed mycobacterial vaccines led to the assumption that bacterial viability was essential for protective immunity. Classical studies demonstrated that inactivated mycobacteria can induce delayed-type hypersensitivity but fail to generate transferable protective T-cell responses, likely because they lack antigens produced by metabolically active organisms [64]. Within this context, BCG killed by extended freeze-drying (EFD-BCG) challenges the traditional distinction between live and killed vaccines. By preserving mycobacterial ultrastructure without thermal denaturation, EFD-BCG retains immunologically relevant structural components and functions as a non-replicating immunomodulatory scaffold capable of shaping immune architecture rather than serving as an inert antigenic preparation.

Across multiple experimental systems, EFD-BCG induces a reproducible immunoregulatory profile distinct from both live and conventionally inactivated BCG. In murine models of airway inflammation and other inflammatory diseases, EFD-BCG promotes plasmacytoid dendritic cell (pDC)-dependent expansion of Forkhead box P3 (Foxp3)^+^ regulatory T cells (Tregs) while suppressing T helper 2 (Th2)- and T helper 17 (Th17)-associated immune responses [65,66]. Similar regulatory effects have been observed in models of intestinal inflammation, where EFD-BCG expands IL-10-producing Tregs and activates anti-inflammatory pathways involving retinoid X receptor–peroxisome proliferator-activated receptor-γ (RXR–PPAR-γ) signaling together with suppression of nuclear factor κB (NF-κB) activation [65,66,67]. These regulatory properties extend beyond inflammatory disease models. EFD-BCG attenuates atherosclerosis and autoimmune neuroinflammation through Treg-mediated mechanisms without impairing host immune competence, while early human studies demonstrate preserved IL-10 induction without evidence of broad immunosuppression [66,67,68].

Collectively, these findings position EFD-BCG not as an inferior substitute for live BCG but as a purposefully engineered non-replicating platform capable of promoting regulatory compatibility within mucosal immune environments. From the perspective of immune layering, the critical determinant of vaccine performance is not bacterial viability alone but the capacity of vaccination to coordinate regulatory, innate, and adaptive immune networks in a tissue-specific manner.

### 6.2. Integrating Innate and Adaptive Immune Design

Experimental studies of non-replicating mycobacterial vaccines support the concept that adaptive effector responses can be partially uncoupled from bacterial replication. Classical studies demonstrated that effective T-cell priming depends preferentially on antigens produced by metabolically active mycobacteria, rather than on structural components exposed following bacterial inactivation [64]. Consistent with this concept, BCG culture filtrate proteins (CFP) efficiently condition APCs and promote cytotoxic T-cell responses in the absence of viable bacilli [69,70]. Complementing these adaptive mechanisms, BCG-derived membrane vesicles (MVs) retain potent innate immunostimulatory activity without requiring bacterial replication. By packaging immunologically active lipoproteins and glycolipids within particulate structures, these vesicles promote Toll-like receptor 2 (TLR2)-dependent innate activation and facilitate trained innate immune programming [71].

The mode of antigen delivery provides an additional level of immune regulation. Formulation strategies such as encapsulation preserve antigen integrity while enabling controlled presentation within mucosal tissues, thereby improving both local and systemic immune responses compared with unformulated vaccines [72]. Genetic engineering approaches further expand opportunities for rational vaccine design. For example, recombinant BCG strains expressing chemokines enhance local recruitment and functional conditioning of innate immune cells following mucosal vaccination, thereby strengthening tissue-specific immune responses [36].

Together, these approaches support a modular vaccine design strategy in which distinct immunological functions are optimized independently yet integrated within a common framework of immune layering. Regulatory compatibility may be provided by non-replicating scaffolds such as EFD-BCG, trained immune programming by membrane vesicles, selective adaptive amplification by CFP-derived antigens, and spatially controlled immune activation through optimized delivery systems.

Viewed from this perspective, live BCG should be regarded not as a single indivisible vaccine but as a composite of separable immunological functions that can be rationally recombined to improve safety, tissue specificity, and durability in next-generation mucosal TB vaccines.

### 6.3. Implications for Clinical Translation and Vaccine Evaluation

These design principles have important implications for translational research and clinical evaluation. Conventional immune correlates derived from peripheral blood capture only a subset of the mechanisms governing mucosal protection and may fail to reflect tissue-localized immune regulation, trained innate immune programming, and tissue-resident memory. The immune-layering framework therefore highlights the need for tissue-informed endpoints that integrate epithelial compatibility, regulatory homeostasis, trained innate immunity, and tissue-resident immune populations alongside conventional systemic immune measures.

Human clinical studies reinforce these limitations. Although BCG revaccination induces measurable antigen-specific immune responses, these responses do not consistently translate into sustained protection against Mtb infection [17]. Host heterogeneity further influences the coordination of regulatory, innate, and adaptive immune layers following mucosal vaccination. Variability in microbiota composition, environmental exposure, and baseline immune regulation is therefore likely to contribute to differences in vaccine responsiveness. Rather than representing experimental noise, this heterogeneity may provide important insights into the tissue-specific regulatory environments that determine effective protection.

The broader tuberculosis vaccine field provides additional support for tissue-informed evaluation strategies. Clinical development of vaccine candidates such as M72/AS01E [73] and H56:IC31 [74] has highlighted the limitations of relying solely on conventional systemic correlates and has reinforced the need to identify immune signatures associated with durable protection. Likewise, recent systems immunology studies of BCG vaccination have demonstrated that protection is associated with complex transcriptional and immunological programs rather than with single immune parameters [8,9]. Together, these observations support the use of integrated immunological frameworks capable of capturing both systemic and tissue-localized immune responses and further emphasize the value of evaluating vaccine-induced immune organization across multiple layers of host defense.

Together, these considerations indicate that the evaluation of next-generation mucosal BCG vaccines should move beyond conventional systemic immune correlates toward integrated tissue-level assessment frameworks that capture both immune function and compatibility with the local tissue environment. Such approaches are likely to provide more informative correlates of durable mucosal protection and facilitate the rational clinical development of future BCG-based vaccines.

### 6.4. Safety Considerations and Clinical Challenges of Mucosal BCG Vaccination

Despite its immunological advantages, mucosal BCG vaccination presents important safety challenges that must be addressed before widespread clinical implementation. Historical experience contributed significantly to the abandonment of oral BCG vaccination, largely because of inconsistent vaccine uptake, variable immunogenicity, difficulties in dose standardization, and concerns regarding the safety of early vaccine formulations [2,11]. Safety considerations also extend to respiratory mucosal delivery. Experimental studies have demonstrated that intranasal BCG vaccination can enhance pulmonary protection while simultaneously inducing dose-dependent inflammatory pathology within the lung [19]. These findings highlight the need to balance protective immunity with the preservation of tissue integrity, particularly in a highly sensitive organ such as the respiratory tract. Excessive activation of trained innate immunity or tissue-resident immune populations may increase the risk of immunopathology even when protective responses are enhanced. Clinical translation further requires consideration of vulnerable populations. Because live BCG is a replicating organism, mucosal administration may pose additional concerns in immunocompromised individuals, including those with primary immunodeficiencies, advanced HIV infection, or receiving immunosuppressive therapy [75]. Although severe adverse events remain uncommon, the potential for disseminated BCG infection necessitates careful risk assessment, monitoring, and patient selection. The safety profile is also likely to differ among mucosal delivery routes. Intranasal, intratracheal, aerosol, and oral administration expose distinct anatomical compartments and may therefore differ in local reactogenicity, inflammatory responses, and tolerability. Future development of mucosal BCG-based vaccines will require optimization of dose, formulation, route of administration, and patient selection to achieve an appropriate balance between immunogenicity, protective efficacy, and safety. Within the immune-layering framework, safety should be viewed not simply as the absence of adverse events but as the successful integration of protective immunity with tissue-specific regulatory mechanisms that preserve physiological homeostasis. Consequently, evaluation of next-generation mucosal tuberculosis vaccines should incorporate safety and tissue-compatibility endpoints alongside conventional immunogenicity measures.

## 7. Conclusions

Several challenges and limitations should be acknowledged when interpreting the evidence discussed in this review. The current literature on mucosal BCG vaccination encompasses diverse animal models, human populations, vaccine formulations, routes of administration, and immunological endpoints, limiting direct comparisons across studies and the generalizability of individual findings. In addition, many mechanistic insights into T_RM_ cells, trained innate immunity, and epithelial–immune interactions have been derived primarily from preclinical studies, and their relative contributions to protection in humans remain incompletely defined. Furthermore, although the immune-layering framework proposed here provides an integrative perspective on protective immunity, many of the relationships among its components are supported by associative rather than causal evidence. These limitations underscore the need for standardized immunological methodologies, longitudinal tissue-based investigations, and well-controlled clinical studies to validate the proposed framework and establish robust correlates of durable mucosal protection.

Evidence from studies of mucosal BCG vaccination demonstrates that durable protection at barrier surfaces cannot be explained solely by the magnitude of systemic immune responses or by the activation of individual immune mechanisms. Rather, protection against TB emerges through coordinated immune layering, in which epithelial regulation, trained innate immunity, tissue-resident adaptive memory, regulatory homeostasis, and systemic immune support are integrated across spatial and temporal scales according to the route and context of vaccination. This framework helps explain why mucosal BCG vaccination elicits immunological programs that are qualitatively distinct from those induced by parenteral vaccination while also clarifying the intrinsic limitations in the durability and anatomical distribution of protection. Human studies consistently demonstrate that different routes of BCG administration generate distinct transcriptional and immunological signatures rather than simply amplifying a common immune response [8,9]. Consequently, effective immunity at the respiratory interface is best viewed as a dynamic, tissue-adapted equilibrium shaped by epithelial regulation, microbial influences, and the local tissue environment. Within this perspective, T_RM_ cells represent a critical—but not exclusive—component of protective immunity. Their establishment and persistence depend on coordinated interactions among local antigen presentation, epithelial instruction, trained innate conditioning, and supportive tissue niches. Earlier studies of heat-killed mycobacteria similarly illustrate that immune activation alone is insufficient for protection, as these preparations induce measurable immune responses yet fail to generate protective T cells capable of mediating adoptive transfer of immunity [64].

The immune-layering framework also provides a conceptual foundation for vaccine redesign. Non-replicating platforms such as EFD-BCG demonstrate that regulatory and innate immune pathways can be engaged without bacterial replication, provided that structural integrity and appropriate immunological signaling are preserved [65,66,67,68]. Together with complementary approaches—including membrane vesicles that promote trained innate immunity and culture filtrate proteins that enhance adaptive immune responses [69,70,71]—these findings support a modular strategy in which distinct immunological functions can be combined to optimize safety, tissue specificity, and durable protection. Clinical studies further emphasize the importance of this integrated perspective. Although BCG vaccination induces measurable immune reprogramming, protection remains incomplete and context dependent [17,29,52], indicating that durable vaccine efficacy depends on coordinating immune responses with tissue-specific regulatory and physiological constraints rather than simply maximizing immunogenicity.

In summary, studies of mucosal BCG vaccination support a shift from reductionist correlates of protection toward tissue-informed models of coordinated immune organization. Framing protective immunity in terms of coordinated immune layering not only provides a mechanistic explanation for variability in BCG efficacy but also offers a conceptual blueprint for the rational design, evaluation, and clinical translation of next-generation mucosal TB vaccines. More broadly, these principles may inform the development of vaccines against other pathogens that initiate infection at mucosal surfaces.

## Figures and Tables

**Table 1:** Key route-dependent differences in immune responses following parenteral and mucosal BCG vaccination.

Parameter	Parenteral BCG Vaccination	Mucosal BCG Vaccination
Primary site of immune induction	Systemic priming in blood and secondary lymphoid tissues	Local priming at the respiratory mucosa, aligned with the site of Mtb entry
Immune organization	Predominantly systemic and circulating immune responses	Spatially localized immunity within the lung and airways
Innate immune programming	Trained immunity is induced, primarily reflected in circulating monocytes	Enhanced local trained immunity within mucosal and resident myeloid compartments
Adaptive memory	Circulating antigen-specific T cells with limited residency in the lung	Robust tissue-resident memory T cells enabling rapid local recall responses
Early pulmonary control	Reduced early control because of spatial separation from the infection site	Enhanced early containment through rapid local immune activation
Durability of protection	Variable; often inferred from systemic immune correlates	Early advantages may not fully translate into durable pulmonary protection
Epithelial and local networks	Limited engagement at the time of vaccination	Direct engagement of epithelial sensing and local regulatory networks
Risk–benefit	Standardized delivery with a low risk of local immunopathology	Greater local efficacy but requiring careful control to avoid pulmonary pathology
Implications for vaccine design	Supports predominantly systemic vaccine paradigms	Highlights vaccination route as a determinant of spatially organized immune layering

**Table 2:** Proposed comparative effects of distinct mucosal BCG delivery routes within the proposed immune-layering framework.

Immune Layer/Parameter	Intranasal	Intratracheal	Aerosol	Oral
Primary site of immune engagement	Upper and lower respiratory mucosa	Lower respiratory tract and lung parenchyma	Broad respiratory tract	Gastrointestinal mucosa
Epithelial regulatory layer	Strong airway epithelial sensing and cytokine induction	Direct epithelial conditioning within the lung	Widespread respiratory epithelial activation	Predominantly intestinal epithelial conditioning
Trained innate layer	Lung macrophage and DC conditioning	Extensive alveolar macrophage exposure and conditioning	Potentially broad pulmonary innate immune reprogramming	Indirect modulation through gut–lung axis signaling
Adaptive resident layer	Efficient induction of airway and lung T_RM_ cells	Efficient pulmonary T_RM_ cell establishment	Potentially broad respiratory T_RM_ cell responses	Limited direct pulmonary T_RM_ cell induction
Systemic support layer	Moderate systemic immune activation	Moderate systemic immune activation	Moderate-to-high systemic immune activation	Interaction with gut-associated immune networks and systemic immune regulation
Potential advantages	Simple administration; efficient local immune induction	Direct targeting of the lung	Broad respiratory tract coverage; clinically scalable	Non-invasive; engages the gut–lung axis
Major limitations	Risk of local inflammation or pulmonary pathology	Invasive delivery procedure	Dose standardization and delivery challenges	Variable uptake and inconsistent immunogenicity
Relevance to immune layering	Predominant engagement of epithelial and immune resident layers	Predominant engagement of innate and resident immune layers	Broad engagement of multiple immune layers	Prominent regulatory and systemic immune modulation

The relative effects summarized in this table represent a conceptual synthesis of the current evidence discussed in this review. They are intended to illustrate the proposed immune-layering framework rather than provide direct quantitative comparisons among mucosal BCG delivery routes.

**Table 3:** Proposed operational definitions, candidate biomarkers, and assessment strategies for the immune layers induced by mucosal BCG vaccination.

Immune Layer	Operational Definition	Candidate Biomarkers/Readouts	Experimental or Clinical Assessment	Interpretation
Epithelial regulatory layer	Local epithelial sensing and regulatory conditioning at the respiratory interface	Epithelial-derived cytokines and chemokines; TGF-β-associated signaling; antimicrobial peptides; epithelial barrier markers; tissue injury and repair markers	Airway or bronchoalveolar lavage (BAL) sampling; epithelial transcriptomics; cytokine profiling; epithelial cell stimulation assays	Defines the local tissue context that calibrates immune activation, preserves barrier integrity, and regulates inflammatory homeostasis
Trained innate layer	Epigenetically and metabolically reprogrammed myeloid compartment with enhanced innate recall responsiveness	H3K4me3 and H3K27ac enrichment; mTOR and HIF-1α pathway activation; glycolytic reprogramming; enhanced TNF, IL-1β, and IL-6 production following secondary stimulation	Monocyte, macrophage, or dendritic-cell restimulation assays; ATAC-seq; ChIP-seq; metabolomics; single-cell transcriptomic or epigenomic profiling	Indicates enhanced innate recall capacity and early antimicrobial preparedness
Adaptive resident layer	Tissue-localized antigen-specific tissue-resident memory T (T_RM_) cell compartment positioned for rapid recall at the respiratory interface	CD69, CD103, CXCR6, antigen-specific CD4^+^/CD8^+^ TRM cells; selected PD-1^+^KLRG1^−^ CD4^+^ TRM subsets; local IFN-γ, TNF, and IL-17 recall responses	Lung tissue or BAL sampling; flow cytometry; single-cell RNA sequencing/T-cell receptor sequencing; antigen-specific stimulation assays	Reflects rapid local adaptive recall responses and tissue-positioned protection against pulmonary Mtb infection
Systemic support layer	Circulating immune components that reinforce local protection and limit extrapulmonary dissemination	Circulating antigen-specific CD4^+^ and CD8^+^ T cells; trained monocytes; IFN-γ, IL-2, and TNF responses; antibody profiles; systemic transcriptional signatures	Peripheral blood immunophenotyping; whole-blood stimulation assays; interferon-γ release assay (IGRA)-like assays; serum proteomics; transcriptomic profiling	Captures systemic immune support that complements local mucosal immunity and contributes to protection beyond the lung
Integrated regulatory outcome	Coordinated balance between protective immune activation, durability, and preservation of pulmonary tissue homeostasis	IL-10; TGF-β; regulatory T-cell frequency; inflammatory pathology scores; tissue injury markers; pulmonary bacterial burden; extrapulmonary dissemination	Longitudinal preclinical studies; imaging; histopathology; microbiological burden assessment; clinical safety and efficacy endpoints	Determines whether coordinated immune activation is protective, durable, and compatible with pulmonary tissue integrity

Candidate biomarkers and assessment strategies are proposed operational criteria derived from the immune-layering framework presented in this review. They are intended to facilitate experimental evaluation and should not be interpreted as validated correlates of protection. Rather, they represent measurable hypotheses for investigating coordinated epithelial, innate, adaptive resident, and systemic immune responses following mucosal BCG vaccination. Abbreviations: ATAC-seq, assay for transposase-accessible chromatin using sequencing; BAL, bronchoalveolar lavage; ChIP-seq, chromatin immunoprecipitation sequencing; HIF-1α, hypoxia-inducible factor-1α; H3K4me3, histone H3 lysine 4 trimethylation; H3K27ac, histone H3 lysine 27 acetylation; IFN, interferon; IGRA, interferon-γ release assay; IL, interleukin; mTOR, mechanistic target of rapamycin; Mtb, *Mycobacterium tuberculosis*; TGF-β, transforming growth factor-β; TNF, tumor necrosis factor; T_RM_, tissue-resident memory.

## Data Availability

No new data were created or analyzed in this study. Data sharing is not applicable to this article.

## Abbreviations

The following abbreviations are used in this manuscript:

APCs	Antigen-presenting cells
BCG	Bacille Calmette–Guérin
CFP	Culture filtrate protein(s)
DC	Dendritic cell
DURTs	Donor-unrestricted T-cells
EFD-BCG	Extended freeze-dried Bacille Calmette–Guérin
Foxp3	Forkhead box P3
GM-CSF	Granulocyte-macrophage colony-stimulating factor
HIF-1α	Hypoxia-inducible factor 1 alpha
HIV	Human immunodeficiency virus
IFN-γ	Interferon gamma
IL	Interleukin
KLRG1	Killer cell lectin-like receptor G1
MAIT	Mucosal-associated invariant T cell
mTOR	Mechanistic target of rapamycin
Mtb	*Mycobacterium tuberculosis*
MV	Membrane vesicle(s)
NK	Natural killer
NF-κB	Nuclear factor kappa B
PD-1	Programmed cell death protein 1
PPAR-γ	Peroxisome proliferator-activated receptor gamma
RXR	Retinoid X receptor
TB	Tuberculosis
TGF-β	Transforming growth factor beta
Th	T helper
TLR2	Toll-like receptor 2
TNF	Tumor necrosis factor
Treg	Regulatory T cell
T_RM_	Tissue-resident memory T cell
